# Editorial: Low anterior resection syndrome—treatment possibilities

**DOI:** 10.3389/fsurg.2025.1565239

**Published:** 2025-03-11

**Authors:** Michail Klimovskij, Audrius Dulskas

**Affiliations:** ^1^ Department of Surgery, East Sussex Healthcare NHS Trust, Saint Leonards-on-Sea, United Kingdom; ^2^ Department of Surgery, National Cancer Institute (Lithuania), Vilnius, Lithuania

**Keywords:** LARS, low anterior resection syndrome (LARS), rectal cancer surgery, robotic rectal cancer surgery, functional outcomes after rectal cancer surgery

**Editorial on the Research Topic**
Low anterior resection syndrome—treatment possibilities

If you ask ChatGPT to tell you about LARS, you receive a fairly comprehensive answer on the etymology and origin of a Scandinavian name. Although anecdotal, it is a good reflection on the perception of and common knowledge on low anterior resection syndrome. Specialists encounter much controversy surrounding this syndrome. Healthcare professionals themselves do not regularly encounter this group of patients and even the patients have only a general understanding of the syndrome.

In lieu of a universal program to educate the public sector on the LARS abbreviation and what it stands for, this research project aims to highlight the controversies, assumptions, and challenges faced when dealing with low anterior resection syndrome.

With the evolution of surgery prioritizing a minimally invasive approach, and the introduction and development of robotics in the colorectal field, rectal-sparing surgery is now the standard compared to what would once have been a Hartmann's or abdominoperineal resection. The surgery removes the need of living with a “stoma bag”, improving quality of life; however, as a downside, this cohort of patients has a tendency to develop LARS to some degree.

The primary aim of this Research Topic was to gain a better understanding of LARS, with a specific emphasis on exploring new etiological theories and potential surgical treatments. Interest was focused on clinical studies that shed light on various topics related to this syndrome, including the efficiency of different treatment modalities and their impact on patients' quality of life.

Part of our research topic aimed to highlight studies investigating the prediction of LARS. One study discusses predictive models such as POLARS and the relevance of predicted outcomes compared to observed outcomes. The paper focused on comparing multicenter results of postoperative LARS with POLARS scores. The scores, however, have shown limited ability to capture the LARS cohort of patients compared to the LARS definition suggested in the International consensus definition of low anterior resection syndrome from 2020. Known assumptions such as “the lower the tumor the worse the function”, neoadjuvant treatment—radiotherapy to pelvis, and ileostomy and the timing of its closure are basic components of prognostic factors used to develop predictive modeling of low anterior resection syndrome (Wang et al.).

All pathologies in healthcare settings are coded to allow systemization, enabling health statistical algorithms and adequate allocation of funding. Surprisingly, LARS does not yet have its own International Classification of Diseases code; this problem is reflected in the paper attached: (Fick et al.).

One of the important factors of low anterior resection syndrome (LARS) treatment is self-management, which requires patient engagement. Colorectal surgeons and nurses may use patient-generated health data (PGHD) to help guide patients in their use of self-management strategies for LARS. However, the perspectives of LARS experts on the use of PGHD remain largely unexplored. The study aimed to explore the perspectives and experiences of LARS experts regarding the use of PGHD in the management of LARS. (Monton et al.).

Currently, the treatment of LARS has no unified approach. There are different modalities treating this complex syndrome, the pathogenesis of which is not yet fully agreed upon. One study in this Research Topic aimed to evaluate the clinical and cost-effectiveness of Transanal irrigation (TAI) or Sacral neuromodulation (SNM) vs. optimized conservative management (OCM) for people with major LARS. Part of this research aimed to review algorithm pathways of studies to learn from the results of different management options, for example transanal irrigation (Klimovskij et al.).

The conclusions of our research remain as an open discussion for anyone interested. This interest is valid as the number of LARS patients is increasing. It is important for patients to speak up and seek help, knowing that this syndrome is recognized, anticipated, and treatable.

It is important for healthcare professionals to know that such a syndrome exists and, if not able to treat it or even know the basic principles of its treatment, refer the patient to an appropriate specialist.

Where we are with LARS today? That is a great question. The aim of our research is to raise awareness of this syndrome, with a focus on deep learning, investment, formulating formalized guidelines on diagnosis and management, and improving the overall recognition of LARS.

We sincerely hope this Research Topic will aid in making that change.

